# Case Control Polysomnographic Studies of Sleep Disorders in Parkinson's Disease

**DOI:** 10.1371/journal.pone.0022511

**Published:** 2011-07-22

**Authors:** Ming-Hui Yong, Stephanie Fook-Chong, Ratnagopal Pavanni, Li-Ling Lim, Eng-King Tan

**Affiliations:** 1 Department of Neurology, National Neuroscience Institute, Singapore; 2 Department of Clinical Research, Singapore General Hospital, Singapore; 3 Duke-NUS Graduate Medical School, Singapore; University of Pennsylvania School of Medicine, United States of America

## Abstract

**Background:**

The relationship between a number of primary sleep disorders and Parkinson's disease (PD) is still debated. There are limited case control polysomnographic studies in PD and most of these study sample sizes are small.

**Methodology/Findings:**

We conducted one of the largest case-control studies involving overnight polysomnographic evaluation, with prospective recruitment of unselected Parkinson's disease patients and healthy controls from an Asian population. The cases were recruited from the specialized movement disorder outpatient clinics in a tertiary referral center, and controls from the same geographical locations. All subjects underwent an overnight polysomnographic study and a multiple sleep latency test.

A total of 124 subjects including 56 patients and 68 controls frequency-matched for age and sex were included. Multivariate analysis revealed that patients had significantly shorter total sleep time than controls (p = 0.01), lower sleep efficiency (p = 0.001) and increased REM latency (p = 0.007). In patients, multivariate analysis showed that reduced total sleep time was significantly associated with increased age (p = 0.001) and increased levodopa dose (p = 0.032). The mean Insomnia Severity Index was higher in PD patients (9.0±7.1) compared to controls (3.3±3.9, p<0.001). The mean Epworth Sleepiness Scale score was higher in PD patients (9.3±5.9 vs. 5.7±4.8, p<0.001). Nocturnal arousals, obstructive sleep apnea, periodic leg movements and objective abnormal sleepiness were not increased in our patients.

**Conclusions/Significance:**

Our case-control polysomnographic study, the first-ever performed in an Asian population, revealed altered sleep architecture and reduced sleep in PD patients compared to controls. Reduced total sleep time was associated with increased age and levodopa dose. However, nocturnal arousals, primary sleep disorders and abnormal sleepiness were not increased in our PD patients suggesting that ethnic/genetic differences may be a factor in the pathophysiology of these conditions.

## Introduction

Sleep disturbances are common in Parkinson's disease (PD) and adversely affect the quality of life [1], [2]. Nocturnal sleep disturbances affecting PD patients include insomnia, sleep disordered breathing (such as obstructive sleep apnea (OSA)), sleep-related movement disorders (restless legs syndrome (RLS), periodic limb movements of sleep (PLMS)) and rapid eye movement (REM) sleep behaviour disorder [1]–[3]. PD patients also experience excessive daytime sleepiness (EDS) and this could be a result of nocturnal disturbances, dopaminergic medications, depression and hypersomnia of central origin not secondary to nocturnal disturbances [2]–[5]. However, the interactions between PD and sleep are complex because many of the sleep related manifestations are not specific to PD.

Overnight polysomnography (PSG) is an objective method of measuring parameters of sleep architecture and pathophysiological events during sleep [6]. It is the gold standard for diagnosing and evaluating the severity of sleep disorders such as sleep related breathing disorders and PLMS [7]–[8]. The Multiple Sleep Latency Test (MSLT) following overnight PSG is also the standard barometer for objective evaluation of EDS and narcolepsy [9].

To date, only nine PSG case control studies in PD have been reported [10]–[18], six of which had a small sample size (n≤30) (case or control arm) [10]–[15], and three of which used historical data which prevented prospective matching [11], [16]–[17]. There was a selection bias for subjects referred for PSG in two of these studies [11], [16]. In addition, only two studies examined the full spectrum of nocturnal sleep on overnight PSG and excessive daytime sleepiness on MSLT, both of which had small sample size in at least one arm [10], [14]. Four of the overnight PSG-only studies focused on sleep apnea in PD [12], [16]–[18] and one focused on REM behaviour disorder [11]. There have been no case control PSG studies in Asia and the role of ethnic/genetic differences in sleep related problems in PD and the link between primary sleep disorders and PD is still unclear.

To address the limitations in the current body of literature, we conducted one of the largest case-control studies involving overnight PSG followed by MSLT, with prospective recruitment of unselected PD patients and healthy controls from an Asian population. We aimed to assess the entire spectrum of sleep disturbances with no specific *a priori* hypothesis (for any sleep disorder), both subjectively using clinical questionnaires and objectively on PSG.

## Methods

A total of 124 subjects including 56 patients diagnosed with idiopathic PD and 68 controls frequency-matched for age (±5 years) and sex participated. The PD cases were recruited from the specialized movement disorder outpatient clinics in a tertiary referral center. Controls were healthy volunteers without chronic debilitating or terminal illness recruited from the same geographical locations and from advertisements in the mass media. As diabetes mellitus, hyperlipidemia and hypertension are common asymptomatic conditions in our general population, these were not excluded. The participation rate was 80% in each arm. All subjects were compensated for their transportation expenses. Written informed consent was taken from all study participants. The work was approved by the Singapore General Hospital and SingHealth Ethics Review Board.

### Data collection and assessment

All subjects provided demographic data and completed a questionnaire screening for symptoms of sleep disorders (insomnia, daytime sleepiness, obstructive sleep apnea, narcolepsy, restless legs syndrome and other sleep related movement disorders). Height, weight and neck circumference were measured, and the body mass index (BMI) calculated for each subject. PD patients provided information on their disease duration and use of dopaminergic medications, and underwent further clinical assessment using Part III (motor section) of the Unified Parkinson's Disease Rating Scale (mUPDRS) and modified Hoehn and Yahr (H&Y) staging. The levodopa equivalent daily dose (LEDD) was calculated according to the standardized formulae as: LEDD (mg/day)  =  [L-dopa (mg)] + [Controlled release L-dopa (mg) ×0.75] + [pramipexole (mg) ×100] + [ropinirole (mg) ×20] + [piribedil (mg)] + [bromocriptine (mg) ×10] [19].

The subjects were also assessed with clinical scales to evaluate subjective sleep disturbance: Insomnia was evaluated using the Insomnia Severity Index (ISI): A higher ISI score reflects higher probability of insomnia, with scores of 15 and above indicative of clinical insomnia (of at least moderate severity). Excessive daytime sleepiness was assessed using the Epworth Sleepiness Scale (ESS), with a score of ≥10 indicating abnormal daytime sleepiness. Subjects also completed the Beck Depression Inventory (BDI): A higher score reflects a greater degree of depressive symptoms.

### Polysomnographic study

Complete PSG was performed with a digital sleep system using the international 10–20 electrode placement for recording C3-A2, C4-A1, O1-A2, O2-A1 electroencephalogram (EEG), electrooculogram (EOG), chin electromyogram (EMG), electrocardiogram (ECG), respiratory effort (from thoracic and abdominal respiratory movements), oximetry, body position, airflow (nasal pressure transducer and thermistor), snoring sound, pulse rate and limb movement channels. Thresholds for abnormal sleep parameters were obtained from the 2^nd^ edition of the International Classification of Sleep Disorders (ICSD-2, 2005) [20]. PSG scoring followed the Rechtschaffen and Kales scoring rules (1968) [21].

### Sleep disordered breathing

Sleep disordered breathing (SDB) was defined as an apnea-hypopnea index (AHI) of 5/hr and above. Severity of SDB was classified as mild (AHI 5–15/hr), moderate (AHI 15.1–30/hr) or severe (AHI >30/hr).

### Periodic limb movements of sleep

Significant PLMS was defined as a periodic limb movement index (PLMI) exceeding 15/hr.

### REM sleep behavior disorder (RBD)

RBD was defined as the presence of REM sleep without atonia (RWA) (excessive sustained or intermittent muscle activity on chin EMG, or excessive phasic twitching on chin or limb EMG during REM sleep) with a history of injurious, potentially injurious or disruptive behaviour associated with sleep and/or documentation of abnormal behaviors observed on time synchronized infrared video recording during those periods of RWA.

### Multiple Sleep Latency Test

Each subject underwent four or five 20-minute opportunities to sleep (nap) at 2 hour intervals. For each nap, the subject was allowed 20 minutes to fall asleep. The time taken to fall asleep was measured, and the average of all naps taken to obtain the mean sleep latency. After falling asleep, the subject would be awoken after 15 minutes.

### Abnormal daytime sleepiness

Abnormal daytime sleepiness was defined if a subject has mean sleep latency of less than 8 minutes.

### Statistical analysis

For univariate analysis, the Mann-Whitney U test was used to compare continuous/ordinal variables and Fisher's exact test to compare frequencies between PD patients and controls. Univariate correlation between variables was performed using the Spearman rank correlation. Multivariate analysis was performed using stepwise multiple linear regression (for continuous dependent variables) and logistic regression (for binary dependent variables). The threshold for significance was set at p<0.05. Analyses were performed using the SPSS Statistics (version 17.0; Chicago: SPSS Inc) software.

## Results

### Demographics

The mean age was 59.3±9.1 years in the control group, and 65.4±9.1 years in the PD patient group. 55.9% of the controls and 60.7% of PD patients were male. The mean BMI was 23.9±3.8 kg/m^2^ in controls, and 22.8±3.7 kg/m^2^ in PD patients. The mean neck circumference was 36.2 ±3.9 cm in controls and 36.8±3.4 cm in PD patients. There was no significant difference in comorbidities (hypertension, hyperlipidemia, diabetes mellitus) between the two groups (Table 1).

**Table 1:** Demographics and Parkinson's disease (PD) patient characteristics.

Demographics	Controls (n = 68)	PD (n = 56)
Age (years)	59.3±9.1 [41, 85]	65.4±9.1 [36, 83]
Gender		
Male	55.9%	60.7%
Female	44.1%	39.3%
BMI (kg/m^2^)	23.9±3.8 [17.9, 35.9]	22.8±3.7 [15.6, 32.1]
Neck circumference (cm)	36.2±3.9 [21.5, 46.0]	36.8±3.4 [30.0, 43.0]
Comorbidities		
Hypertension	26.5%	32.1%
Hyperlipidemia	27.9%	30.4%
Diabetes mellitus	7.4%	12.5%
Use of a benzodiazepine	0.0%	19.6%
Characteristics of PD patients		
Disease duration (years)		6.4±4.1 [1], [19]
Modified Hoehn & Yahr stage		2.5 [1], [4]
mUPDRS		21.5±11.8 [5, 62]
Medications		
Levodopa equivalent daily dose (mg/day)		409.4±265.1 [0, 1100]
Levodopa		83.9%
DA		21.4%
Levodopa only		75.0%
DA only		12.5%
Levodopa and DA		8.9%
Neither levodopa nor DA		3.6%

BMI: body mass index, DA: dopamine agonist.

All continuous values were expressed as mean ± SD [min, max] except for Hoehn & Yahr stage of PD patients, which was expressed as median [min, max].

PD patients had mean disease duration of 6.4±4.1 years. The median modified Hoehn and Yahr (H&Y) stage was 2.5. 11 patients (19.6%) were in H&Y stages 1–1.5, 23 patients (41.1%) in stages 2–2.5, 18 patients (32.1%) in stage 3 and 3 patients (5.4%) in stage 4. The mean mUPDRS score was 21.5±11.8. The mean levodopa equivalent daily dose (LEDD) was 409.4±265.1 mg/day. 75.0% of the PD patients were on levodopa alone, 12.5% on dopamine agonists alone, 8.9% on both levodopa and dopamine agonists and 3.6% on neither levodopa nor dopamine agonists. For the patients on dopamine agonists, the mean dopamine agonist dose (in levodopa-equivalent units) was 72.1±36.5 mg/day. 19.6% of PD patients were on a benzodiazepine.

PD patients had more depression than controls as reflected by significantly higher BDI scores (10.2±7.7 vs. 3.3±3.4, p<0.001 after accounting for the effect of age and sex using multiple linear regression). 2 PD patients were on antidepressants (escitalopram and fluoxetine).

### Subjective sleep related complaints

PD patients reported more complaints of insomnia (difficulty falling asleep, difficulty staying asleep/frequent awakenings, unrefreshing sleep), excessive daytime sleepiness, abnormal movements in sleep and sleep talking than controls (p<0.001) (see Table 2).

**Table 2:** Percentage of controls and PD patients with individual sleep complaints.

Sleep complaint	Controls(n = 68)	PD patients(n = 56)	*p*-value(Fisher's exact test)	*p*-value (Multivariate analysis^a^)
Difficulty falling asleep	4.4%	41.1%	<0.001	<0.001
Difficulty staying asleep	5.9%	46.4%	<0.001	<0.001
Unrefreshing sleep	2.9%	39.3%	<0.001	<0.001
Snoring	50.0%	67.9%	0.067	0.053
Excessive daytime sleepiness	2.9%	66.1%	<0.001	<0.001
Abnormal movements in sleep	7.4%	46.4%	<0.001	<0.001
Acting out dreams	0.0%	30.4%	<0.001	-
Sleep talking	5.9%	51.8%	<0.001	<0.001

a Forward (Wald) stepwise logistic regression taking into account effects of age and sex.

### Nocturnal sleep disturbances

Multivariate analysis accounting for any effect of age showed that the mean ISI was significantly higher in PD patients (9.0±7.1, range 0 to 27) compared to controls (3.3±3.9, range 0 to 16) (p<0.001). 21.4% of PD patients were interpreted to have clinical insomnia (ISI score ≥15) compared to 1.5% of controls (p = 0.006). For PD patients, we evaluated the association between the ISI score and age, gender, disease duration, H&Y stage, mUPDRS score, LEDD, use of dopamine agonists and degree of depression. Increased ISI in PD patients was significantly correlated with increased depressive symptoms measured with the BDI (p = 0.003), and multivariate analysis confirmed this significant effect of BDI score on ISI (p = 0.001).

### Polysomnographic study

The overnight PSG and MSLT results are shown in Table 3. PD patients had significantly shorter total sleep time than controls (p = 0.01), lower sleep efficiency (p = 0.001) and increased REM latency (p = 0.007). Percentage of sleep spent in stage 1 was higher in PD patients (p = 0.017), while that of REM stage was reduced in PD patients compared to controls (p<0.001). The arousal index (AI) did not differ between PD patients and controls (p = 0.506).

**Table 3:** PSG and MSLT sleep parameters and sleep disorders present in study subjects.

Sleep parameter	Controls	PD	*p*-value(Univariate analysis^a^)	*p*-value(Multivariate analysis^b^)
Total sleep time (min)	340.2±84.6	277.1±104.4	<0.001	0.010
Sleep efficiency (%)	76.6±18.3	59.4±22.0	<0.001	0.001
Sleep onset latency (min)	19.2±29.1	40.3±68.6	0.082	0.133
REM latency (min)	128.2±79.8	177.0±104.1	0.013	0.007
% stage 1 sleep	13.0±8.8	21.3±16.7	0.010	0.017
% stage 2 sleep	48.0±11.2	46.0±16.4	0.665	0.849
% deep sleep	21.6±10.7	23.1±17.3	0.881	0.466
% REM sleep	17.1±6.9	8.5±7.4	<0.001	<0.001
Arousal index (/hr)	13.4±8.5	12.9±8.5	0.898	0.506
PLMI (/hr)	10.7±21.5	10.3±18.1	0.595	0.549
AHI (/hr)	12.2±13.1	12.5±15.6	0.302	0.999
MSL (min)	9.5±4.2	12.5±5.6	0.002	0.010
*Presence of sleep disorders*				
Significant PLMS	20.9%	26.4%	0.519	0.478
OSA	65.7%	49.1%	0.062	0.043
RBD	0%	0%	-	-
Abnormal daytime sleepiness	39.4%	23.6%	0.080	0.208

AHI: Apnea-hypopnea index, PLMI: periodic limb movements index, MSL: mean sleep latency, PLMS: periodic limb movements of sleep, OSA: obstructive sleep apnea, RBD: REM sleep behaviour disorder, RLS: restless legs syndrome.

Continuous variables are expressed as mean ± SD.

a Mann-Whitney U test was used to compare continuous variables, and Exact Fisher's test to compare frequencies

b Stepwise multiple linear regression was used for continuous variables and forward (Wald) stepwise logistic regression for binary outcomes (presence of each sleep disorder). Multivariate analysis took into account any effects of age and sex (and in the case of OSA and AHI, any effect of BMI and neck circumference).

N.B. Overnight PSG reports for 3 PD patients and 1 control, and MSLT reports for 1 PD patient and 2 controls were missing. They were not included in the relevant analyses.

In PD patients, multivariate analysis determined that reduced total sleep time was significantly associated with increased age (p = 0.001) and increased LEDD (p = 0.032). Reduced sleep efficiency was associated with increased age (p = 0.002) and increased modified H&Y stage (p = 0.031). BDI score was not significantly associated with either total sleep time or sleep efficiency despite being associated with subjective insomnia. Percentage of sleep in REM stage correlated negatively with age (p = 0.02) but on multivariate analysis no significant factor was found to be associated with REM stage sleep percentage. None of the PSG parameters were found to have a significant association with disease duration. AI did not correlate significantly with age, disease severity or other sleep parameters.

### Periodic limb movements

There was no significant difference between the PLMI between PD patients (10.3±18.1/hr) and controls (10.7±21.5/hr) (p = 0.549). 26.4% of PD patients and 20.9% of controls had significant PLM (PLMI ≥15) (p = 0.478). In PD patients, PLMI was not significantly associated with age, gender, disease duration, H&Y stage, mUPDRS, LEDD, or ESS score. None of these factors could predict for the presence of significant PLM in PD patients.

### Sleep related breathing disorders

There was no significant difference in the AHI between PD patients (12.5±15.6/hr) and controls (12.2±13.1/hr) (p = 0.999). The mean central apnea index (CAI) was extremely low and clinically insignificant in both groups (0.68±4.7/hr in PD patients, and 0.22±0.61/hr in controls). 49.1% of PD patients had OSA compared to 65.7% of controls. 15.1% of the PD patients had mild OSA, 18.9% had moderate OSA, and 15.1% had severe OSA. In controls 37.3% had mild OSA, 19.4% had moderate OSA and 9.0% had severe OSA. 96.2% and 38.4% of PD patients with OSA reported snoring and feeling unrefreshed on waking from sleep respectively, and 40% had abnormal ESS scores. On MSLT 26.9% of PD patients with OSA had abnormal daytime sleepiness. Snoring was the only significant predictor of OSA in the multivariate analysis (p = 0.001).

### Relation of subjective poor nocturnal sleep to overnight PSG parameters in PD patients

In PD patients, increased ISI was significantly correlated with reduced sleep efficiency (p = 0.019). It also correlated with reduced total sleep time (p = 0.07). There was no significant correlation between the ISI and the sleep onset latency, stages of sleep, AI, AHI or PLMI.

### REM sleep behaviour disorder (RBD)

53 PD patients and 60 controls underwent a clinical assessment by a sleep neurologist for sleep disorders. Of these, 22.6% of the PD patients but 0% of controls provided a clinical history suggesting possible REM behaviour disorder. However, the diagnosis of RBD in these subjects could not be confirmed: On overnight PSG there was no evidence of RBD for all of our subjects.

### Restless legs syndrome

5.7% (n = 3) of PD patients and 1.7% (n = 1) of controls who underwent the clinical assessment were diagnosed to have RLS (p = 0.252). Of these 4 subjects, 1 PD patient with RLS also experienced significant PLMS on overnight PSG (PLMI ≥15).

### Daytime sleepiness

The mean ESS score was significantly higher in PD patients (9.3±5.9, range 0 to 23) than in controls (5.7±4.8, range 0 to 21) (p<0.001), with 41.1% of PD patients having an abnormally high ESS score (≥10) compared to 19.1% of controls (p = 0.01). In patients, ESS score was significantly correlated with H&Y stage (p = 0.033), mUPDRS score (p = 0.025), LEDD (p = 0.041), BDI score (p = 0.002) and ISI score (p = 0.021). After multivariate analysis, BDI score was found to be the only factor significantly associated with ESS score (p = 0.01).

### Multiple Sleep Latency Test

The mean sleep latency (MSL) was lower in controls (9.5±4.2 min) than PD patients (12.5±5.6 min) (p = 0.010). Abnormal daytime sleepiness (MSL <8 min) was found in 39.4% of controls and 23.6% of PD patients (p = 0.208). 16.7% of controls and 12.7% of PD patients had severe excessive daytime sleepiness (MSL <5 min) (p = 0.544). No sleep onset REM periods were observed in any of the naps of PD patients. Of the 13 PD patients with abnormal daytime sleepiness, 9 had a primary sleep disorder on overnight PSG: 6 patients had OSA, 2 patients had abnormal PLM and 1 patient had both OSA and abnormal PLM. For PD patients, mean sleep latency was not significantly associated with age, gender, disease duration, H&Y stage, mUPDRS, LEDD or dopamine use.

### Gender differences in sleep parameters on PSG and MSLT

We compared the sleep stages between men and women within each group. In each group, there was no significant difference in age between men and women. In the control group, gender differences in sleep architecture were in line with that reported in the literature [22]: percentage of sleep time spent in stage 1 was increased in men compared to women (15.3±10.1% vs. 10.2±5.8%, p = 0.013) and deep sleep percentage was reduced in men (18.6±10.2% for men vs. 25.3±10.4% for women, p = 0.009). These trends did not seem to be disrupted in our PD group: Deep sleep percentage was reduced in men compared to women (20.6±19.7 vs. 27.0±12.5) (p = 0.039). Percentage of sleep in stage 1 was higher in men than women (25.7±19.1 vs. 14.6±9.0), but this only almost reached significance (p = 0.069).

### Effect of medications on sleep in PD patients

Comparisons between PD patients on levodopa treatment alone and PD patients on dopamine agonist treatment alone showed a significant difference only in stage 2 sleep – this was increased in patients on agonist only (43.2±15.5% vs. 61.3±15.3%, p = 0.009), but not other PSG parameters.

## Discussion

We conducted a comprehensive case control PSG study to evaluate a wide spectrum of sleep symptoms in PD. Our study demonstrated that PD patients experience poorer nocturnal sleep quality, as manifested by a greater frequency of sleep complaints and a higher degree of insomnia measured on the ISI. More severe insomnia in PD patients was supported by overnight PSG findings of lower total sleep time and sleep efficiency in PD patients than controls, but there was no increase in arousals or primary sleep disorders on PSG (OSA, PLMS and RBD) to suggest these as dominant causes of reduced nocturnal sleep in PD patients, and parameters measuring these disorders (AI, AHI, PLMI) also did not correlate significantly with the ISI, total sleep time or sleep efficiency. Altered sleep architecture compared to controls (increased REM latency, reduced percentage of sleep time spent in REM stage and increased stage 1 sleep compared to controls) was found in PD patients but no significant effect of this on the ISI was found.

Our data suggest that certain clinical factors were associated with nocturnal sleep disturbances. PD patients with more depressive symptoms had greater subjective insomnia, although there was no significant association between BDI and reduced total sleep time or sleep efficiency. Reduced total sleep time was associated with increased age and LEDD while reduced sleep efficiency was associated with increased age and H&Y stage. The poorer sleep efficiency seen with increased H&Y stage may be due to increased motor disability and, in part, higher LEDD in patients with more severe PD, or reflect progressive intrinsic pathology of PD affecting sleep centres in the brain. A negative correlation between total sleep time and levodopa dose has also been previously reported by Happe et al. (2005) [13]. Diederich et al. (2009) did not find a correlation between LEDD and total sleep time or percentage of sleep spent in deep sleep; however they found an increased proportion of patients with relatively high deep sleep percentage in the patient group with an intermediate LEDD range [23]. The effects of dopaminergic drugs on sleep are known to be dose dependent, with lower doses promoting deep and REM sleep and reducing wakefulness, and higher doses having the opposite effect, possibly due to differential activation of D1 and D2 receptors [24]. Our results imply that dosage of dopaminergic treatment to improve total sleep time in PD patients suffering from nocturnal motor symptoms needs to be adjusted to balance the benefit from relief of these symptoms and the alerting doperminergic effect at higher doses. Controlled release levodopa or dopamine agonists are commonly suggested to achieve improved sleep in PD patients by relieving nocturnal motor symptoms such as akinesia[13], [24]–[27], though it is not well established if they improve total sleep time: Van den Kerchove et al. (1993) found a reduction in total time awake with sustained-release levodopa [24], [26] and Stocchi et al. (1998) reported an increase in sleep time with chronic-release levodopa/carbidopa that only almost reached significance [28], while Wailke et al. (2011) did not find an increase in total sleep time with controlled-release levodopa/carbidopa [15]. It is not certain if individualized optimization of levodopa dosage for each patient in such studies would help to improve total sleep time.

Optimizing subjective sleep quality also involves management of depressive symptoms in PD patients, and sedative antidepressants taken in the evening may help improve nocturnal sleep in PD patients with insomnia [27].

PD patients also experienced more severe daytime sleepiness subjectively as measured by the ESS than controls. Interestingly, this was not supported objectively by our MSLT results; there was no significant difference between the proportion of PD patients with abnormal sleepiness and that of controls. Again, increased depressive symptoms significantly affected PD patients' perception of daytime sleepiness on the ESS scale, and treatment of these symptoms may be helpful. Although subjective sleepiness measured by ESS was positively correlated with subjective insomnia (ISI), total sleep time, sleep efficiency and other measured parameters on overnight PSG were not significantly associated with increased ESS score. Although levodopa and dopamine agonists are regarded as contributors to daytime sleepiness, there was no correlation between LEDD and MSL. There were no significant differences in mean ESS score or MSL detected between PD patients on dopamine agonist treatment and those on levodopa treatment only, although only 21.4% of our PD patients were taking dopamine agonists. Rye et al. (2000) also found no correlation between MSL and levodopa daily dose [10]. Arnulf et al. (2002) found only a weak correlation between MSL and levodopa daily dose, but no correlation with dopamine agonist daily dose or LEDD in PD patients with daytime sleepiness [29].

The prevalence of OSA and AHI was not increased in the PD group. PD patients with OSA had reduced deep and REM sleep compared to PD patients without OSA, however the presence of OSA (and AHI) did not affect the total sleep time, sleep efficiency, subjective insomnia, or daytime sleepiness experienced by PD patients. Other PSG studies by Cochen de Cock et al. (2010), Trotti et al. (2010) and Diederich et al. (2005) found no increased prevalence and severity of, or increased impact on sleep by sleep apnea in PD patients [16]–[18]. On the other hand, Shpirer et al. (2006) and Maria et al. (2003) found increased prevalence and severity of sleep apnea [12], [14].

None of the PD patients in our study were observed to have RBD on overnight PSG, while prevalences of 16–50% have been reported in previous PSG studies [11], [30], [31]. RBD may not manifest daily and it is still possible that some of the patients in our study did have RBD, evidence of which we were not able to capture with only one night of PSG.

Other hypothesized relationships between PD and altered sleep architecture have not been consistently proven in PSG studies either. Diederich et al. (2005) previously reported that changes in nocturnal sleep architecture are progressive with disease duration, with findings that total sleep time, REM sleep time and sleep efficiency decreased as disease duration increased [32]. However, in our study we did not find a significant association between disease duration and the sleep parameters on overnight PSG or MSLT. Happe S et al. (2005) also did not find any correlation between overnight PSG parameters and disease duration [13]. In previous studies on excessive daytime sleepiness, Rye et al. (2000), Arnulf I et al. (2002) and Poryazova et al. (2010) did not find an association between reduced MSL and disease duration [10], [29], [33]. In addition, we and Shpirer et al. (2006) did not find a significant decrease in percentage of time spent in deep sleep in PD patients compared to controls as was reported by Wailke et al. (2011) [14], [15]. Diederich et al. (2005) found increased, rather than decreased, deep sleep time in PD patients compared to age-, sex- and AHI-matched controls [16]. Methodological differences (retrospective vs. prospective recruitment and sources of controls) as well as differences in patient characteristics and medications may partly explain variances in published studies.

Genetic influences on circadian rhythm and various sleep disorders are being increasingly recognized [34], and may be an additional factor accounting for the differences between our prevalence of primary sleep disorders and that found in the Western literature. This is illustrated by the discovery of genetic variants that increased the risk of RLS within an Icelandic population [35], [36], and the much lower prevalence of RLS in Asian populations compared to Caucasian populations [37]. Genes influencing craniofacial morphology, obesity, pharyngeal muscle control of the upper airway and ventilator control, together with environmental influences may affect risk for the development of OSA [34], [38].

Our study has limitations. We evaluated one night of PSG and one day of MSLT, as such our results may have been influenced by a first night effect and any day-to-day variation of sleep disturbances in our subjects. However, this is the standard practice for the evaluation of sleep disorders, and applied to both PD patients and controls, as such a fair comparison between the two groups could still be made. Also, differences in study design and PD patient profile (with regards to disease duration, disease severity and medication dosages) compared to other published studies prevent us from attributing differences in our findings entirely to genetic differences in the Asian population. Lastly, as we recruited PD patients with different stages of disease, our study does not distinguish between sleep dysfunction that affects all PD patients as a whole and that which affects only PD patients at a particular stage of disease.

In conclusion, our case control PSG study, the first-ever performed in an Asian population, showed altered sleep architecture and reduced sleep on overnight PSG in PD patients compared to controls. Reduced total sleep time was associated with increased age and levodopa dose. However, unlike some smaller studies in Caucasians, nocturnal arousals, primary sleep disorders and abnormal daytime sleepiness were not increased in our PD patients, suggesting that ethnic/genetic differences may be a factor in the pathophysiology of these conditions. Treating depressive symptoms and judicious adjustment of dopaminergic medications may be useful in managing subjective sleep-related complaints in PD patients.
